# The Influence of Quercetin on Maternal Immunity, Oxidative Stress, and Inflammation in Mice with Exposure of Fine Particulate Matter during Gestation

**DOI:** 10.3390/ijerph14060592

**Published:** 2017-06-02

**Authors:** Wei Liu, Minjia Zhang, Jinqiu Feng, Aiqin Fan, Yalin Zhou, Yajun Xu

**Affiliations:** 1 Department of Nutrition and Food Hygiene, School of Public Health, Peking University, Beijing 100191, China; liuwei19560912@163.com (W.L.); minjiazh@163.com (M.Z.); fengjinqiu1992@163.com (J.F.); fanaiqin1990@163.com (A.F.); zhouyalin2017@163.com (Y.Z.); 2 Beijing Key Laboratory of Toxicological Research and Risk Assessment for Food Safety, Peking University, Beijing 100191, China

**Keywords:** PM_2.5_, pregnancy, quercetin, lymphocyte subset, inflammation, oxidative stress

## Abstract

The objective is to investigate the influence of PM_2.5_ exposure on peripheral blood lymphocyte subsets in pregnant mice and the antagonism of quercetin on adverse effects induced by PM_2.5_ exposure. Pregnant mice were randomly divided into control group, PM_2.5_ model group and 3 quercetin intervention groups. Dams in all groups except the control group were exposed to PM_2.5_ suspension by intratracheal instillation on gestational day (GD) 3, 6, 9, 12 and 15. Meanwhile, each dam was given 0.15% carboxymethylcellulose sodium (CMCS) (control group & PM_2.5_ model group) and different doses of quercetin (quercetin intervention groups) by gavage once a day from GD0 to GD17. The percentage of lymphocyte subsets, Biomarkers of systemic inflammation injuries (IL-2, IL-6, IL-8 & TNF-α) and oxidative stress indicators (CAT, GSH & HO-1) in peripheral blood of the dams were analyzed. The number of T cells increased, accompanied by increased level of IL-2, IL-6, IL-8 and HO-1 due to PM_2.5_ exposure. Less CD4+ and CD8+ T cells were counted in 100 mg/kg quercetin intervention group, compared with PM_2.5_ model group. Quercetin may inhibit cytokine production, especially in IL-6 and IL-8 and may upgrade the level of HO-1. Our findings indicate that PM_2.5_ could significantly influence the distribution of T-lymphocyte subsets, activate inflammatory reaction and elevate oxidative stress level in peripheral blood of pregnant mice. Certain dose of quercetin administration during pregnancy may protect the dams against the adverse effects through various ways.

## 1. Introduction

Fine particulate matter 2.5 (PM_2.5_, aerodynamic diameter ≤ 2.5 μm) is associated with diseases such as type 1 diabetes [1] and asthma [2]. It is also one of the leading risk factors for premature mortality [3]. As a carrier, PM_2.5_ may absorb various potentially harmful molecules such as organic molecules, transition metals, reactive gases, microbial components, and minerals [4]. In addition, the composition and concentration of PM_2.5_ mixture varies dramatically in different regions and seasons. For example, concentrations of its components, including chlorine, zinc, and bromide, have been found to be higher during winter.

Pregnancy is a complex, sophisticated physiological process. The potential impact of environmental stimuli on maternal immune function is directly related to fetal development [5]. Delius demonstrated that PM_2.5_ could influence macrophage activation state and the possible enhancement of T cell proliferation [6]. The research carried out by Herr et al. showed that the increase of CD3+ and CD4+ lymphocytes percentages in cord blood was significantly associated with PM_2.5_ exposure during early gestation, and PM_2.5_ in ambient air may influence fetal immune development via shifts in cord blood lymphocytes distributions [7].

It is reported that PM_2.5_ may cause many kinds of diseases, while the mechanisms still remain unclear. High oxidative stress [8] and inflammatory response [9], two biologically linked processes [10], are considered to be potential mechanisms based on previous studies. PM exposure is a known risk factor for the release of inflammatory factors [10,11] and the occurrence of systemic oxidative stress in humans [12,13]. Especially during the pregnancy period increased levels of oxidative stress can be expected [14]. One study showed that particulate air pollution exposure in early life plays a role in increasing systemic oxidative stress, at the level of the mitochondria, both in mother and fetus [15]. Many phytochemicals in foods have the extremely important function of anti-oxidation and anti-inflammation. Quercetin, a herbal flavonoid, may be derived from a variety of plants, such as apple, onion, tea, strawberries, and broccoli. It has been identified to possess various biological activities, such as anti-oxidation [16], antimicrobial activity [17], wound-healing [18], anti-cancer activity [19], and immune-modulatory activity [20]. Zhong et al. found that higher flavonoid intake could attenuate adverse cardiac autonomic effects induced by PM_2.5_ exposure in elderly men [21]. At the molecular level, quercetin inhibits oxidant stress through inducing heme oxygenase-1 (HO-1) and reduces oxidative damage via modulating the expression of antioxidant genes [22].

As far as we know now, few studies have investigated the changes of lymphocyte subsets during pregnancy with PM_2.5_ exposure, and even less attention has been paid to the antagonism function of quercetin against the adverse effects caused by PM_2.5_ on pregnant females. The objective of this study is to clarify the effect of quercetin intake on maternal immunity responses induced by PM_2.5_ exposure.

## 2. Materials and Methods

### 2.1. Preparation of PM_2.5_ and Chemicals

The samples of PM_2.5_ were collected by a particulate sampler (TH-150C, Wuhan Tianhong Instruments Co. Ltd., Wuhan, China) in residential area of Beijing, China, from 1 December 2014 to 20 February 2015. Filters were cut into 1–2 cm^2^ squares. The filter squares were agitated in ultrapure water with an ultrasonic shaker for 20 min 3 times. The solution was filtered through 8 layers of gauze and centrifuged at 12,000 rpm for 20 min. The sediment was collected by a vacuum freeze drier (FDU-1100, Tokyo Rikakikai Co. Ltd., Tokyo, Japan). The dry PM_2.5_ powder was diluted in sterile phosphate-buffered saline (PBS) (0.01 M, pH 7.4) at a concentration of 15 mg/mL and kept at −20 °C before experiments. An extra control sample from unexposed filters was processed identically. Morphology of PM_2.5_ particles was observed with a scanning electron microscope (SEM) (JSM-5600LV, Jeol Ltd., Tokyo, Japan).

Quercetin (Sigma products, purity ≥ 95.0%) was respectively dissolved in 0.15% CMCS at a concentration of 10, 20, and 40 mg/mL. IL-2, IL-6, IL-8, TNF-α, and HO-1 enzyme-linked immunosorbent assay (ELISA) kit were purchased from Freemore (Beijing, China). FITC-anti-CD3, PE/Cy7-anti-CD8, Brilliant Violet 421-anti-CD4, PE-anti-CD5, APC-anti-CD19, and red blood cell lysis buffer were purchased from BioLegend (San Diego, CA, USA).

### 2.2. Animals and Treatment

Specific pathogen-free (SPF) 8-week-old ICR mice were provided by the Department of Laboratory Animal Science of Peking University (Beijing, China, SCXK-2012-0001). The animals were quarantined for 7 days after shipping and were maintained in a temperature- and humidity-controlled animal facility with a 12-h/12-h light/dark cycle (lights on 7:00 a.m.). Mice were provided with basic mouse chow and distilled water ad libitum until pregnancy was confirmed. After the quarantine period, female mice were mated with healthy male mice overnight and were checked for vaginal plugs the next morning at 7:00 a.m. The presence of a vaginal plug signified Gestational Day 0 (GD 0).

The plug-positive females were randomly divided into five groups (ten dams/group): a control group (Group A), a PM_2.5_ model group (Group B), and three quercetin intervention groups (Groups C, D, and E). All dams were individually housed and provided with commercial pregnancy forage for mice and sterile distill water ad libitum until sacrificed. At 9:00–11:00 a.m. on GD 3, 6, 9, 12, and 15, dams were anesthetized with 3% isoflurane after body weight recording and received intratracheal instillation. When we conducted intratracheal instillation, the angle of body restraint we chose was 45 (supine head up), as reported in another paper [23]. Dams in Groups B, C, D, and E received PM_2.5_ samples (15.0 mg/kg), and dams in Group A were treated with same amount of suspension from extracts of unexposed filters. In addition, dams in Groups C, D, and E received 50 mg/kg, 100 mg/kg, or 200 mg/kg quercetin, respectively, at a volume of 0.005 mL/g per day via oral gavage from GD 0 to GD 17. Dams in Groups A and B received an oral gavage of 0.15% CMCS in the same period. Gavage was conducted at 2–4 p.m. every day. All dams were sacrificed by cervical dislocation on GD 18. The use of animals in this research was conducted in compliance with the Guidelines for Animal Research of Peking University (number of animal experimental ethical investigational tab: LA2015111).

The treatment of the dams are listed in Table 1.

Body weight and food consumption were recorded on GD 0, 3, 6, 9, 12, 15, and 18, and the food utilization rates were calculated from the following equation: food utilization rate = weight gain/food consumption × 100%.

### 2.3. Biochemical Analysis of the Maternal Serum

Blood samples were collected from the orbital sinus by removing eyeballs under deep anesthesia. After clotting at room temperature, the blood samples were centrifuged at 3000 rpm for 15 min, then the serum was transferred to new tubes and preserved at −80 °C until analysis. The contents of catalase (CAT) and glutathione (GSH) activity in serum were assayed with a commercial colorimetric assay kit (Nanjing Jiancheng Bioengineering Institute, Jiangsu, China). Interleukin 2 (IL-2), interleukin 6 (IL-6), interleukin 8 (IL-8), tumor necrosis factor α (TNF-α), and heme oxygenase 1 (HO-1) in serum were assayed by ELISA kits, respectively, according to the manufacturer’s instructions.

### 2.4. Organ Index and Lung Histology

After blood sampling, all dams were scarified by cervical dislocation. Spleen and thymus of each dam were separated immediately and we noted the weight after blood was wiped off. Organ indexes (Sx) of these two organs were calculated as Sx = weight of experimental organ (mg)/weight of experimental animal (g).

Lungs of each dam were separated immediately and inflated with 10% buffered formalin, fixed overnight, and embedded in paraffin, sectioned at 5 μm, and stained with hematoxylin and eosin (H&E). Lung samples were analyzed blinded to group assignments, and the assessment of histological lung injury was performed by grading, as Table 2 shows [24].

### 2.5. Flow Cytometric Analysis

Lymphocyte subgroups in peripheral blood were analyzed by a flow cytometer (Gallios, Beckman Coulter, Brea, CA, USA). The blood samples were placed in EDTA containing vacutainer tube before the dams were sacrificed. 50 μL of anticoagulated blood was mixed with anti-mouse mAbs (FITC CD3, PE/Cy7 CD8, Brilliant Violet 421 CD4, PE CD5, and APC CD19) and incubated for 20 min at room temperature in the dark. After first-stage incubation, 1 mL of red blood cell lysis buffer (containing ammonium chloride, potassium carbonate, and EDTA) was added to each blood tube, and was then incubated at room temperature and protected from light for 10 min. Then, the samples were centrifuged at 392× *g* at 25 °C for 5 min, and the supernatant was discarded. The samples were washed with 3 mL of cell staining buffer before being centrifuged at 392× *g* at 25 °C for 5 min, and the supernatant was discarded. The cells were resuspended in cell staining buffer and then analyzed on Gallios.

### 2.6. Statistical Analysis

Values were presented as the mean ± SD. The results were statistically analyzed using SPSS 17.0 software (SPSS, Inc., Chicago, IL, USA). Intergroup differences were analyzed using one-way analysis of variance (ANOVA) followed by the least significant difference (LSD) post-hoc test if equal variance existed, or Tamhane’s T2 post-hoc test if equal variance did not exist. *p* < 0.05 was regarded as statistically significant.

## 3. Results

### 3.1. Morphology of PM_2.5_ Particles

**Figure 1:**
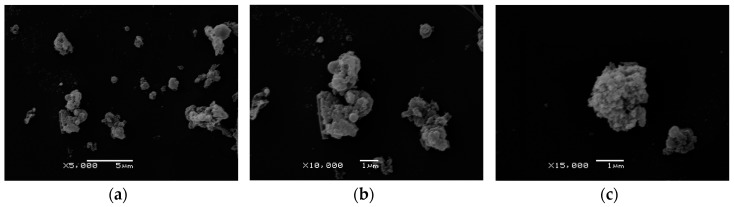
Morphology of PM_2.5_ particles. (**a**) Large area image of PM_2.5_ suspension (5000×); (**b**) Partial area image of PM_2.5_ suspension (10,000×); (**c**) SEM images of particles with rough surface. The scale bars are 5 μm for image (**a**), 1 μm for image (**b**), and 1 μm for image (**c**).

Figure 1 shows the typical SEM picture of PM_2.5_ suspension: the PM_2.5_ exhibited circular, elongated, and irregular shapes. Figure 1c shows the typical example of PM_2.5_ particles with rough surfaces.

### 3.2. Body Weight and Food Utilization

**Figure 2:**
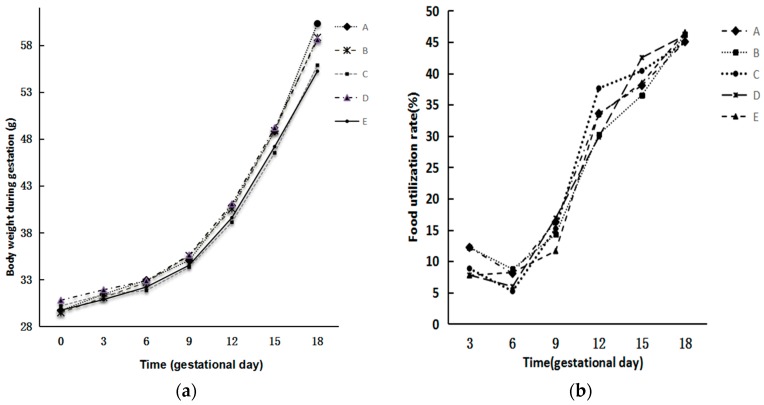
Changes of body weight and food utilization rate of dams in different groups. (**a**) Changes of body weight; (**b**) Changes of food utilization rate. The food utilization rate was calculated from the following equation: food utilization rate = weight gain/food consumption × 100%. There was no significant difference in body weight and food utilization rate among the five groups (*p* > 0.05).

A time-dependent increase in the body weight of the dams was observed in each group, without significant differences in the mean body weight among five groups. There was no significant difference in calculated food utilization rate among them either (*p* > 0.05) (Figure 2).

### 3.3. Histological Lung Injury Score

**Figure 3:**
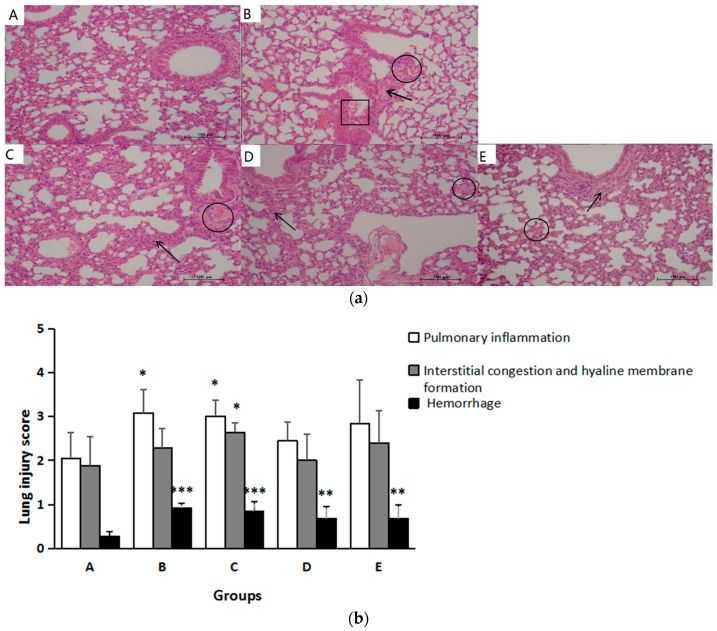
Effects of quercetin on maternal lung structure and injury score with PM_2.5_ exposure during gestation. (**a**) Lung sections were stained with hematoxylin and eosin. Original magnification, ×200. Arrows indicate typical areas with inflammatory cell infiltrates. Circles indicate typical areas with interstitial congestion and hyaline membrane formation. Boxes indicate typical area with hemorrhage; (**b**) Slides were scored by two independent blinded observers for the severity evaluation of lung injury. Histology scores are displayed as mean ± SD. Compared with Group A, * indicates *p* < 0.05, ** indicates *p* < 0.01, *** indicates *p* < 0.001. The analysis of scores of interstitial congestion and hyaline membrane formation and hemorrhage were conducted with “Tamhane’s T2 test”.

Overall lesion scores indicated that PM_2.5_ induced apparent pathology changes in the lung. As shown in Figure 3, obvious inflammatory cellular infiltration was observed in the lungs of dams in Groups B and C (*p* < 0.05). In Groups D and E, inflammatory cell soak was rare. In terms of interstitial congestion and hyaline membrane formation, injury scores in Groups B, D, and E were higher than those in Group A without significant difference (*p* > 0.05). However, Group C had significantly higher scores compared with Group A (*p* < 0.05). Hemorrhage occurred commonly in Groups B, C, D, and E (*p* < 0.05).

### 3.4. Organ Coefficient of Spleen and Thymus

As shown in Table 3, there was no significant difference found in the spleen and thymus coefficient among five groups (*p* > 0.05) (Table 3).

### 3.5. The Lymphocyte Subsets in Peripheral Blood

**Figure 4:**
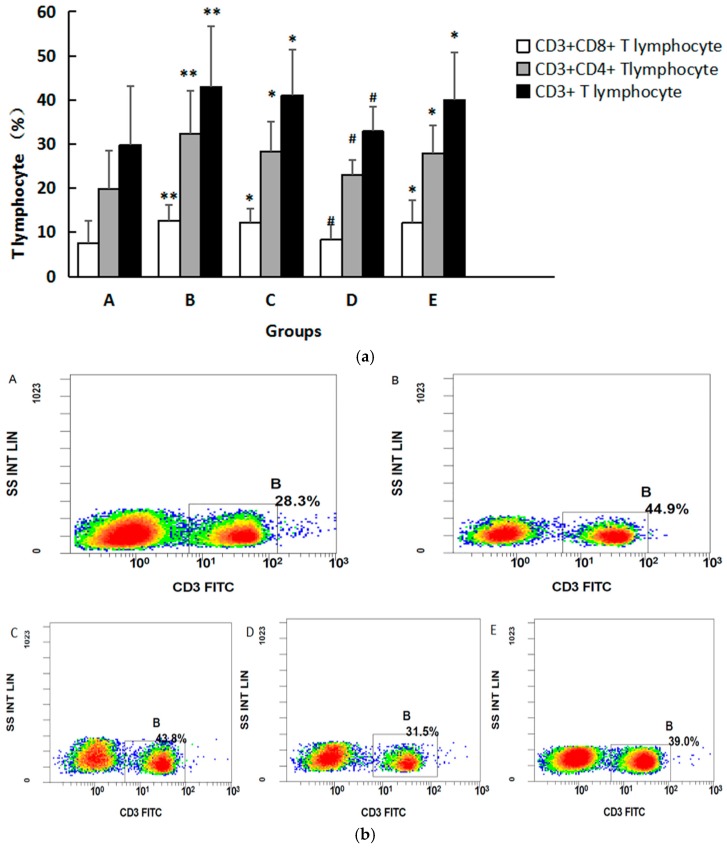

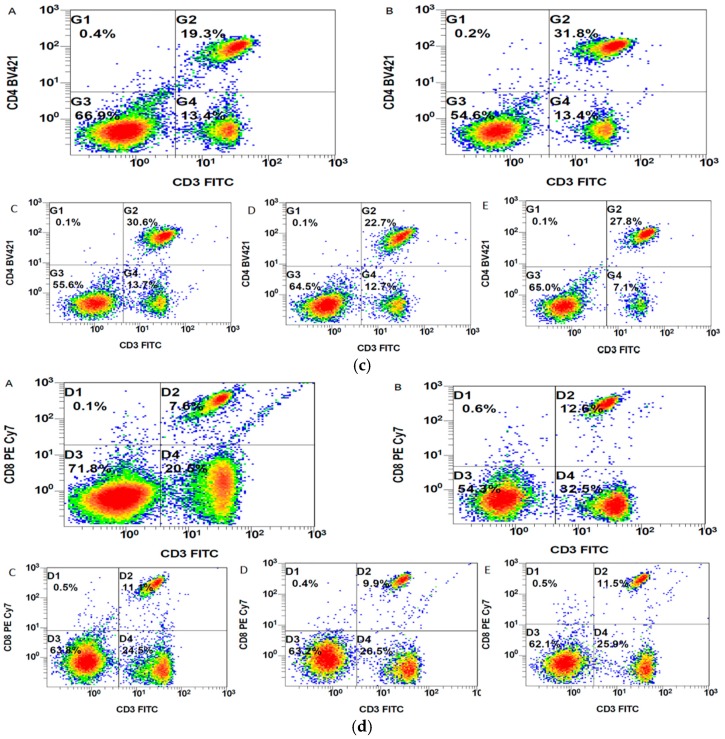
(**a**) Changes of T lymphocytes in serum of mice in different groups. The data are expressed as mean ± SD. Compared with Group A, * indicates *p* < 0.05, ** indicates *p* < 0.01. Compared with Group B, ^#^ indicates *p* < 0.05; (**b**) Representative flow plots showing CD3 staining in cells; (**c**) Representative flow plots showing CD3 and CD4 staining in cells; (**d**) Representative flow plots showing CD3 and CD8 staining in cells.

We found that the percentages of CD3+ cells in Groups B, C, and E were significantly higher than those in Group A (*p* < 0.05). A greater decrease was observed in Group D compared with Group B (*p* < 0.05). CD3+CD4+ cells in Groups B, C, and E were higher than those in Group A (*p* < 0.05), and compared with those in Group B there were fewer CD3+CD4+ in Group D (*p* < 0.05). The percentages of CD3+CD8+ cells in Groups B, C, and E were significantly higher than those in Group A (*p* < 0.05), and compared with Group B there were fewer CD3+CD8+ cells in Group D (*p* < 0.05), as shown in Figure 4a. Representative flow plots are shown in Figure 4b–d. There were no significant differences in CD19+, CD19+CD5+, CD19+CD5- B lymphocyte counts among five groups. (See Table 4).

### 3.6. Serum Cytokines

**Figure 5:**
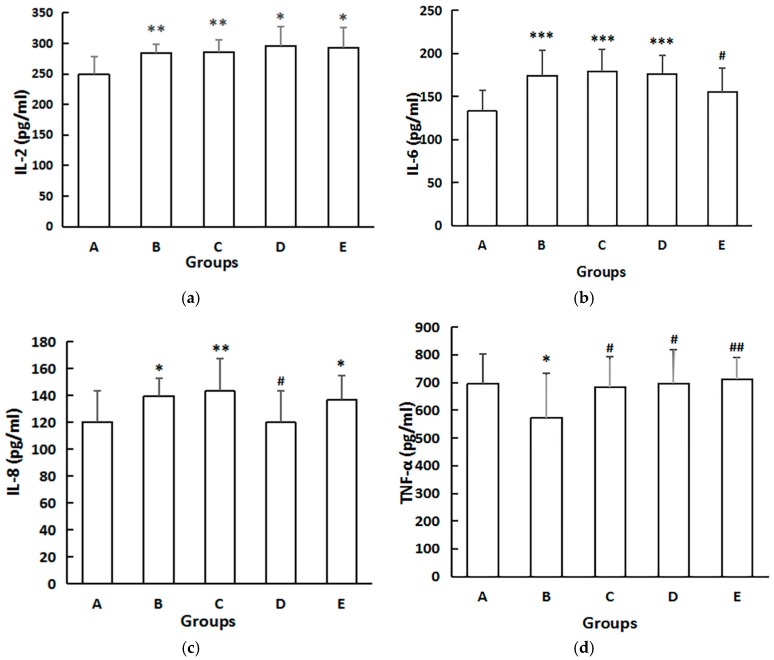
The effect on dams serum concentrations of cytokines. (**a**) Effect on serum IL-2; (**b**) Effect on serum IL-6; (**c**) Effect on serum IL-8; (**d**) Effect on serum TNF-α. The serum levels of IL-2, IL-6, IL-8, and TNF-α in serum were detected by ELISA according to the manual of ELISA kits. The data are expressed as mean ± SD of each group. Compared with Group A, * indicates *p* < 0.05, ** indicates *p* < 0.01, *** indicates *p* < 0.001. Compared with Group B, ^#^ indicates *p* < 0.05, ^##^ indicates *p* < 0.01. The analysis of IL-6 was conducted with “Tamhane’s T2 test”.

As shown in Figure 5, serum levels of IL-2, IL-6, and IL-8 in the dams were all significantly upregulated (*p* < 0.01) in Group B, compared with Group A. IL-6 level was lower in Group E than that in Group B. IL-8 levels were considerably lower in Group D compared with those in Group B (*p* < 0.05). Interestingly, TNF-α levels were considerably lower in Group B compared with those in Group A, and higher in Groups C, D, and E compared with those in Group B (*p* < 0.05) (Figure 4).

### 3.7. Biomarkers of Systemic Oxidative Injuries

**Figure 6:**
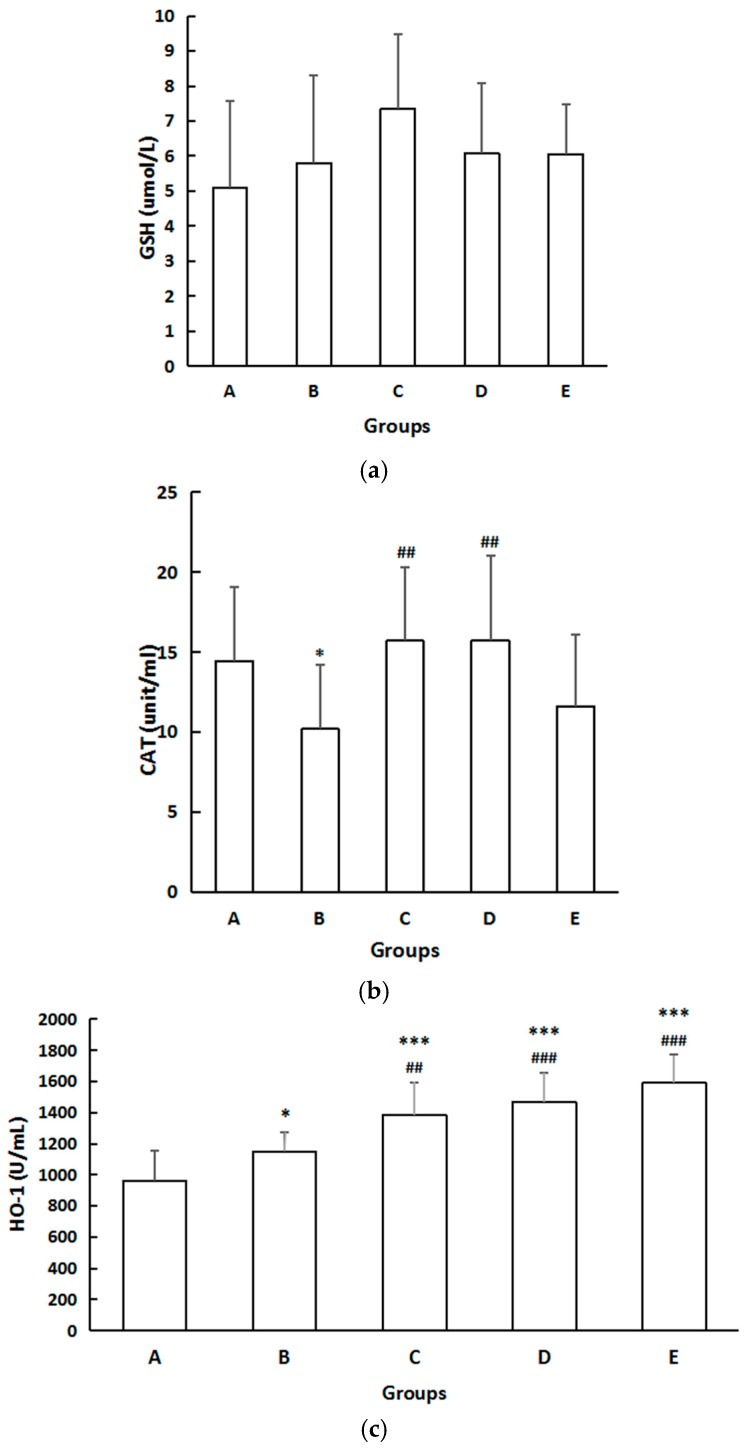
(**a**) The effect on GSH. The data are expressed as mean ± SD of each group. There was no difference in serum GSH among five groups; (**b**) The effect on CAT. The data are expressed as mean ± SD of each group. Compared with Group A, * indicates *p* < 0.05. Compared with Group B, ^##^ indicates *p* < 0.01; (**c**) The effect on concentration of HO-1. The data are expressed as mean ± SD of each group. Compared with Group A, * indicates *p* < 0.05, *** indicates *p* < 0.001. Compared with Group B, ^##^ indicates *p* < 0.01, ^###^ indicates *p* < 0.001.

There were no differences in serum GSH among the five groups (Figure 6). However, the CAT level in Group B decreased significantly compared with that in Group A (*p* < 0.05). Compared with Group B, the serum CAT level increased considerably (*p* < 0.05) in Groups C and D (Figure 6). Concentration of HO-1 in Group B was higher than that of Group A (*p* < 0.05), and compared with Group B, serum HO-1 levels were considerably increased (*p* < 0.05) in Groups C, D, and E (Figure 6).

## 4. Discussion

A growing body of epidemiological evidence indicates that ambient air pollution has adverse effects on pregnant women and fetal development [25,26]. PM_2.5_ could even attribute 3.2 million premature deaths per year, according to the survey conducted by Global Burden of Disease (GBD) [3]. It is widely known that chemical compositions of PM_2.5_ can remarkably influence toxicity. According to a previous study, in which PM_2.5_ collected in the same area, the PM_2.5_ exhibited high densities of O, Si, C, Fe, Ca, Mg, Al, K, and S [27]. Prior reports have suggested that inhaled particulate matter may potentiate innate immune function [6], while the mechanism of PM_2.5_ exposure during pregnancy served as a stimulus for serum T cell activation has not been well described. Maternal immune function changes could extend to lactation or even future, resulting in a long-term impact on health for both mother and her offspring [28].

We used animal models to investigate the influence of PM_2.5_ exposure on maternal immunity, oxidative stress, and inflammation indicators. The intratracheal instillation dosage of PM_2.5_ was determined on the basis of previous researches [13,29] and our pre-experiment. The results of our present research indicated that PM_2.5_ exposure during pregnancy had great impact on T-lymphocyte subsets proportion, serum cytokines, and biomarkers of systemic oxidative injuries in maternal peripheral blood.

The activity of T-lymphocyte subsets is an important indicator of immune homeostasis [30]. A report identified three critical phases of immune development during pregnancy: (1) Weeks 8–10: initiation of hematopoiesis; (2) Weeks 10–16: hematopoietic cell migration and progenitor cell expansion; (3) Week 16–birth: colonization of bone marrow and thymus [31]. On our study, dams were exposed to PM_2.5_ throughout pregnancy. Our research showed that PM_2.5_ exposure during pregnancy may increase the number of CD3+CD4+ and CD3+CD8+ T lymphocytes, breaking the original homeostasis and activating the immunology response. Elevated levels of serum IL-2, IL-6, and IL-8 were also observed in dams, which indicates the activation of severe systemic inflammatory reaction. Liu et al. have released a similar result that a significant increase of serum IL-6 was examined in dams, who were exposed to PM_2.5_ on Day 10 and Day 18 during gestation with the dose of 15 mg/kg [29]. The reason of why serum TNF-α in Group B was lower than that of Group A was still unclear, although Aztatzi-Aguilar et al. also observed that TNF-α level of kidney cortices was decreased in the PM_2.5_ intervention group [32]. Oxidative stress occurred in the dams with PM_2.5_ exposure, as a decreased level of CAT and an increased level of HO-1 were detected significantly. CAT is an anti-oxidant enzyme that converts hydrogen peroxide to water and oxygen and the decreased level of CAT means decreased anti-oxidant capacity. HO-1 is an enzyme that may catalyze the process of degrading heme to generate CO, biliverdin, and free iron [33], playing an important role in immunoregulation and oxidative stress defense [34,35]. The expression of HO-1 in response to oxidative stress suppresses the release of endogenous proinflammatory ligands from injured cells, thus further promoting the process of relieving inflammation and homeostasis reestablishment [35]. It should be noted that, in the PM_2.5_ group, there was increased activity of HO-1, which suggested a self-protection effect against oxidative damage.

It is infeasible to solve PM_2.5_ pollution thoroughly in a short period due to economic and social impact factors, so we hope to boost health against the injury caused by PM_2.5_ through diet intervention in our daily life. Supported by literature, oxidative damage is regarded as one of the mechanisms by which PM_2.5_ contributes to adverse effects on the human body, with the definite mechanisms remaining unclear so far. Quercetin, a common flavone widely found in fruits and vegetables, is a powerful antioxidant and free radical scavenger [36]. It can be acquired from a normal diet, whereas its content is not sufficient enough and the intake varies in different groups of people. Quercetin has been reported to show no maternal or fetal toxicity, even with a daily intake of 2000 mg/kg body weight during gestation in rats [37]. Referring to previous research [38], we determined the following three doses of quercetin: 50, 100, and 200 mg/kg.

Our results indicated that quercetin has a protective effect on lymphocyte subgroups, serum cytokines, and oxidative stress changes under PM_2.5_ exposure. Compared with the model group, the medium-dose quercetin group showed significantly lower percentage of CD3+, CD4+, and CD8+ T-lymphocyte subgroups, further proving that quercetin may improve the immune function in dams. Quercetin also has a pleasurable inhibitory effect on inflammation changes. In quercetin groups, serum levels of IL-6 and IL-8 were close to the control group. We assumed that the release of serum IL-6 and IL-8 were obviously suppressed by quercetin with its anti-inflammatory function. Previous studies have demonstrated that glycosylation of quercetin could enhance the early innate immunity effectively by activating macrophages to secrete TNF-α [20], while the mechanisms involved are largely undefined. This may explain why TNF-α level in quercetin groups were higher than other groups. Quercetin could adjust the oxidative stress state of the body through increasing serum concentration of CAT and HO-1 to reduce the injuries caused by PM_2.5_. Similarly, it has been reported that quercetin may upregulate HO-1 against endotoxic stress through the involvement of MAPKs [39].

**Figure 7:**
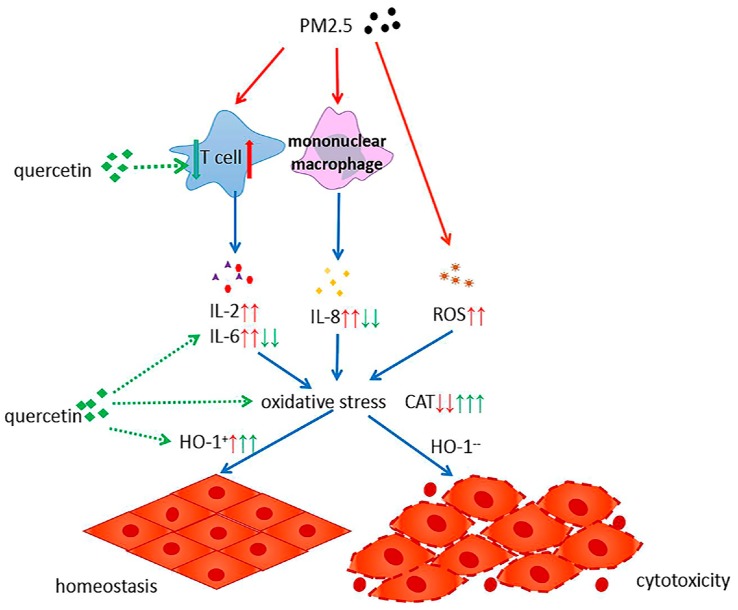
The mechanism of quercetin suppressing the adverse health effects of PM_2.5_. ↑ and ↓ indicates the effects of PM_2.5_; ↑ and ↓ indicates the effects of quercetin.

PM_2.5_ exposure affects the percentage of T-lymphocyte subsets in pregnant mice, with increased inflammatory factors and activated oxidative stress. Our study indicated that quercetin could reduce these adverse effects in multiple ways (Figure 7). In the first place, quercetin may inhibit the proliferation of T lymphocyte. Moreover, quercetin plays an essential role in the process of anti-inflammatory and anti-oxidation to antagonism oxidative stress state caused by PM_2.5_ exposure. Last but not the least, quercetin may increase the expression of HO-1 to promote body homeostasis reestablishment. The recommended dose of quercetin intake is still a controversial issue [40]. Some studies have shown that high-dose polyphenol intervention could result in negative effects [41,42]. A high dosage of quercetin intake during pregnancy was shown to increase iron storage in the liver by upregulating iron-associated cytokine expression like IL-6 and IL-10 [43]. Our experimental results indicated that the intervention effect of the medium-dose quercetin group was the most apparent, meaning that the protective function of quercetin may be displayed within a proper dose range, and higher dose quercetin intake will not yield further improvement.

Although the findings of this study could give new insights to the understanding of changes of lymphocyte subgroups, serum cytokines, and oxidative stress under PM_2.5_ exposure, compared to inhalation, intratracheal instillation could lead to less homogeneous particle distribution. Even though intratracheal instillation is used as an alternative method for studying inhalation exposure, the localization of the test material in the lungs from inhalation and intratracheal instillation differs [23], which may affect the results. In addition, further studies are needed to explore the mechanism of lymphocyte changes under PM exposure.

## 5. Conclusions

PM_2.5_ may significantly influence the proportion of T-lymphocyte subsets, and cause inflammation and oxidative damage. Quercetin may partly attenuate these adverse effects in various ways. Quercetin may inhibit the proliferation of T lymphocyte and has a pleasurable inhibitory effect on inflammation changes. In addition, quercetin plays an essential role in the process of anti-oxidation to antagonism oxidative stress state caused by PM_2.5_ exposure. Taking a proper dose of quercetin as dietary supplements during pregnancy may have beneficial effects on health.

## Figures and Tables

**Table 1:** Animal treatment.

Group	Intervention	N	PM_2.5_ (mg/kg)	Quercetin (mg/kg)
A	Normal control	10	-	-
B	PM_2.5_ model control	10	15	-
C	low-dosage quercetin	10	15	quercetin (50)
D	middle-dosage quercetin	10	15	quercetin (100)
E	high-dosage quercetin	10	15	quercetin (200)

**Table 2:** Histological lung injury score criteria.

**A**	**Pulmonary Inflammation Score Criteria**
0	No lesions
1	Minimal lymphocytic inflammation restricted to perivascular and peribronchiolar regions
2	Moderate perivascular and peribronchiolar inflammation with mildly increased numbers of alveolar macrophages, lymphocytes, and eosinophils
3	Marked perivascular and peribronchiolar inflammation with moderately increased numbers of alveolar macrophages, lymphocytes, eosinophils, and multinucleated giant cells
4	Severe perivascular and peribronchiolar inflammation with markedly increased numbers of alveolar macrophages, eosinophils, lymphocytes and multinucleated giant cells
5	Severe perivascular and peribronchiolar inflammation with effacement of alveolar parenchyma and small airways by sheets of inflammatory cells
**B**	**Interstitial Congestion and Hyaline Membrane Formation**
1	Normal lung
2	Moderate (<25% of lung section)
3	Intermediate (25–50% of lung section)
4	Severe (>50% of lung section)
**C**	**Hemorrhage**
0	Absent
1	Present

**Table 3:** The spleen and thymus indexes (mean ± SD).

Group	Spleen Index (mg/g)	Thymus Index (mg/g)
A	2.09 ± 0.40	1.01 ± 0.15
B	2.04 ± 0.49	1.09 ± 0.18
C	2.21 ± 0.54	1.15 ± 0.22
D	2.18 ± 0.24	1.02 ± 0.17
E	2.18 ± 0.45	1.16 ± 0.26

**Table 4:** The maternal lymphocyte subsets in peripheral blood.

Group	CD3+	CD3+CD4+	CD3+CD8+	CD19+	CD19+CD5−	CD19+CD5+
A	29.82 ± 13.38	19.91 ± 8.60	7.56 ± 5.13	28.56 ± 14.13	97.27 ± 1.71	2.73 ± 1.71
B	42.91 ± 13.70 **	32.29 ± 9.87 **	12.61 ± 3.62 **	31.81 ± 15.67	96.98 ± 1.34	3.02 ± 1.34
C	41.09 ± 10.32 *	28.36 ± 6.70 *	12.17 ± 3.14 *	22.12 ± 7.06	97.68 ± 1.09	2.32 ± 1.09
D	32.86 ± 5.65 ^#^	23.03 ± 3.38 ^#^	8.28 ± 3.43 ^#^	26.24 ± 7.37	97.43 ± 0.57	2.58 ± 0.57
E	39.98 ± 10.71 *	27.86 ± 6.48 *	12.09 ± 5.19 *	23.63 ± 11.55	97.49 ± 0.81	2.53 ± 0.83

Note: compared with Group A, * indicates *p* < 0.05, ** indicates *p* < 0.01; compared with Group B, ^#^ indicates *p* < 0.05. The analysis of CD19+ cell was conducted with “Tamhane’s T2 test”.

## Abbreviations

PM_2.5_	fine particulate matter
PBS	phosphate-buffered saline
GD	gestational day
CMCS	carboxymethyl cellulose sodium
IL-2	interleukin-2
IL-6	interleukin-6
IL-8	interleukin-8
TNF-α	tumor necrosis factor α
CAT	catalase
GSH	glutathione
HO-1	heme oxygenase 1
ROS	reactive oxygen species
PBS	phosphate-buffered saline
SEM	scanning electron microscope
H&E	hematoxylin and eosin
ELISA	enzyme-linked immunosorbent assay
ANOVA	one-way analysis of variance
